# P-290. Clinical and microbiological characteristics of patients infected with ceftazidime/avibactam-resistant *Klebsiella pneumoniae* carbapenemase-producing *K. pneumoniae* strains

**DOI:** 10.1093/ofid/ofae631.493

**Published:** 2025-01-29

**Authors:** Szu-Yu Liu, Sheng-Hua Chou, Chien Chuang, Yu-Chien Ho, Chih-Han Juan, Yi-Tsung Lin

**Affiliations:** Taipei Veterans General Hospital, Taipei, Taipei, Taiwan (Republic of China); Institute of Emergency and Critical Care Medicine, National Yang-Ming University, Taipei, Taiwan, Taipei, Taipei, Taiwan (Republic of China); Taipei Veterans General Hospital and National Yang Ming Chiao Tung University, Taipei, Taipei, Taiwan; Taipei Veterans General Hospital, Taipei, Taipei, Taiwan (Republic of China); Taipei Veterans General Hospital and National Yang Ming Chiao Tung University, Taipei, Taipei, Taiwan; Division of Infectious Diseases, Department of Medicine, Taipei Veterans General Hospital, Taipei, Taiwan, Taipei, Taipei, Taiwan (Republic of China)

## Abstract

**Background:**

*Klebsiella pneumoniae* carbapenemase (KPC)-producing *K. pneumoniae* with ceftazidime/avibactam (CZA) resistance emerged after the widely use of CZA. The most common resistance mechanism was mutations in *bla*_KPC_. We aimed to study the clinical and microbiological characteristics in patients infected with CZA-resistant KPC-producing *K. pneumoniae*, and focus on the comparison between the strains with or without KPC variants (wild-type KPC).

**Methods:**

A retrospective study was conducted in Taipei Veterans General Hospital from February 2019 to July 2023. Hospitalized unique adult patients infected with CZA-resistant KPC-producing *K. pneumoniae* were included. Clinical characteristics and outcomes were collected, and genes encoding carbapenemases were detected by polymerase chain reaction followed by Sanger sequencing.

**Results:**

A total of 44 cases with CZA-resistant KPC-producing *K. pneumoniae* infections were identified, and most of them were critically ill with the median APACHE II score 23.0 (IQR, 19.3-29.8). Pneumonia was the most common infection (n=18). Only 15 patients (34.1%) received appropriate treatment regimens, and tigecycline (n=8) and meropenem-based therapy (n=5) were the common regimens. Twenty-seven strains (61.4%) harbored KPC variants, and a total of 16 different types were identified. KPC-33 (n=10) was the most common, followed by KPC-35 (n=3). The overall 28-day mortality was similar between patients infected with and without KPC variants (22.2% vs 23.5%, *p*=1.000). A previous isolation of carbapenem-resistant *K. pneumoniae* and exposure to CZA were more common in KPC variants group compared with wild-type KPC group (92.6% vs 58.8%, *p*=0.017, 92.6% vs 41.2%, *p*< 0.001, respectively). KPC variants-producing strains also had a higher proportion of CZA MIC > 16 mg/L (85.2% vs 41.2%, *p*=0.002) and restored meropenem susceptibility (MIC≤ 4 mg/L) (70.4% vs 5.9%, *p*< 0.001) than that in wild-type KPC strain.

**Conclusion:**

CZA-resistant KPC variants-producing *K. pneumoniae* has the tendency of restored meropenem susceptibility and higher level of CZA resistance compared with the wild type KPC-producing strain. The optimal therapy in these patients needs further study.

**Disclosures:**

**All Authors**: No reported disclosures

